# Complicated Meckel’s diverticulum due to axial torsion and fibrous band formation: a rare cause of acute abdomen

**DOI:** 10.1093/jscr/rjag605

**Published:** 2026-07-31

**Authors:** Francisco de León Gálvez

**Affiliations:** Private Surgery Clinic, Retalhuleu, Guatemala

**Keywords:** acute abdomen, axial torsion, Meckel’s diverticulum

## Abstract

Acute abdomen is a potentially life-threatening condition requiring prompt evaluation and surgical intervention. Symptomatic Meckel’s diverticulum in adults is uncommon and may mimic other intra-abdominal pathologies. We report a 25-year-old man presenting with diffuse abdominal pain, nausea, diarrhea, and signs of peritoneal irritation suggestive of complicated appendicitis. Exploratory laparotomy revealed axial torsion of a Meckel’s diverticulum with irreversible vascular compromise caused by a fibrous band. Diverticulectomy with wedge resection was performed, resulting in complete recovery. This case highlights the diagnostic challenge of complicated Meckel’s diverticulum and the importance of considering rare causes of acute abdomen.

## Introduction

Meckel’s diverticulum is the most common congenital anomaly of the gastrointestinal tract and results from incomplete obliteration of the omphalomesenteric duct. Its reported incidence ranges from 0.6% to 4% and it is classically associated with the ‘rule of twos,’ describing its prevalence, location, size, and potential presence of ectopic mucosa [1–3].

Although most individuals remain asymptomatic throughout life, ~3.7%–6.4% develop complications requiring medical or surgical treatment. Clinical manifestations are frequently nonspecific and often resemble more common abdominal disorders, including gastroenteritis, peptic ulcer disease, diverticulitis, biliary pathology, and acute appendicitis. Consequently, preoperative diagnosis remains challenging, particularly in adults [4, 5].

The spectrum of complications differs according to age. Gastrointestinal bleeding secondary to ectopic gastric mucosa is more common in children, whereas intestinal obstruction and inflammatory complications predominate in adults. Less frequent presentations include perforation, neoplasia, and torsion [6, 7]. Axial torsion of Meckel’s diverticulum is exceptionally rare and may result in ischemia, gangrene, and generalized peritonitis if left untreated. Because of its rarity and nonspecific presentation, diagnosis is usually established only during surgical exploration.

We present a rare case of axial torsion of Meckel’s diverticulum caused by a fibrous band, producing irreversible vascular compromise and clinical findings suggestive of complicated appendicitis.

## Case report

A 25-year-old male with no relevant medical or surgical history presented with a three-day history of progressively worsening abdominal pain, initially localized to the right iliac fossa and mesogastrium, associated with diarrhea and nausea. Before admission, he received symptomatic outpatient treatment without clinical improvement. On examination, the patient appeared uncomfortable and demonstrated abdominal distension, hyperactive bowel sounds, generalized abdominal tenderness, and signs of peritoneal irritation with abdominal rigidity. Laboratory investigations revealed leukocytosis of 20 000/mm^3^ with neutrophilic predominance and elevated C-reactive protein levels. Hemoglobin concentration, platelet count, coagulation profile, and renal function tests were within normal limits.

Based on the clinical presentation and laboratory findings, complicated acute appendicitis was strongly suspected. Given the presence of generalized peritoneal signs, the patient was taken to the operating room for exploratory laparotomy.

**Figure 1:**
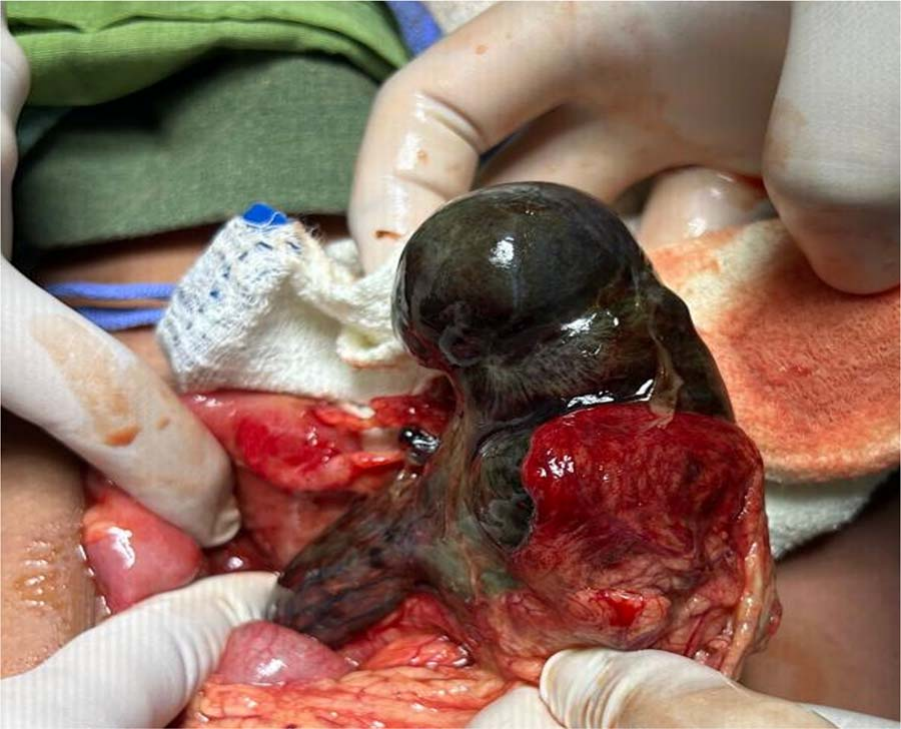
Initial intraoperative visualization of the diverticulum.

**Figure 2:**
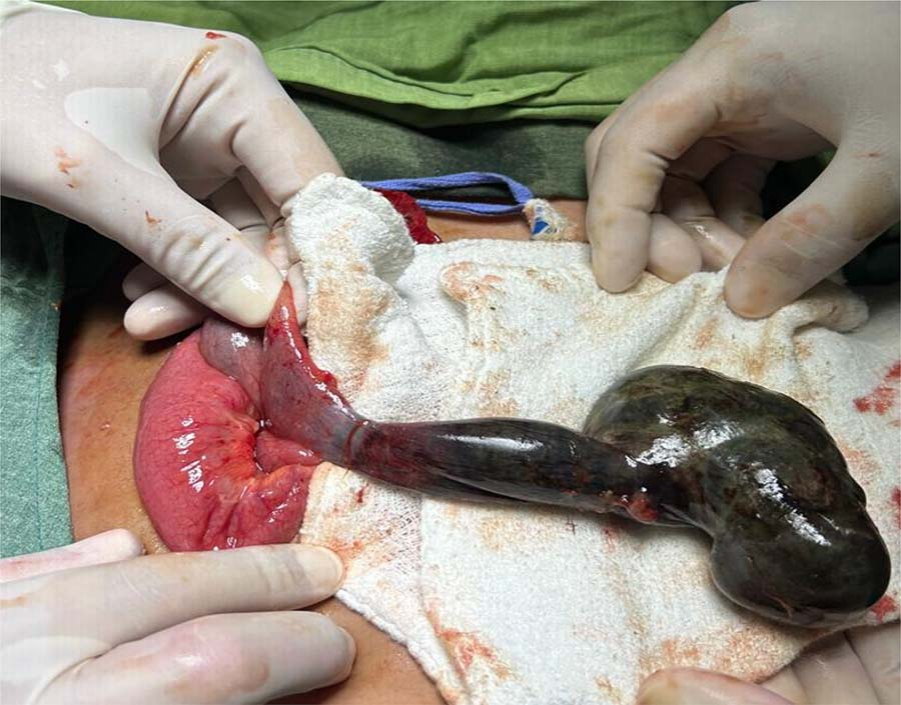
Demonstrating part of the fibrotic band and the area of strangulation and torsion caused by it producing the irreversible vascular compromise of the diverticulum.

**Figure 3:**
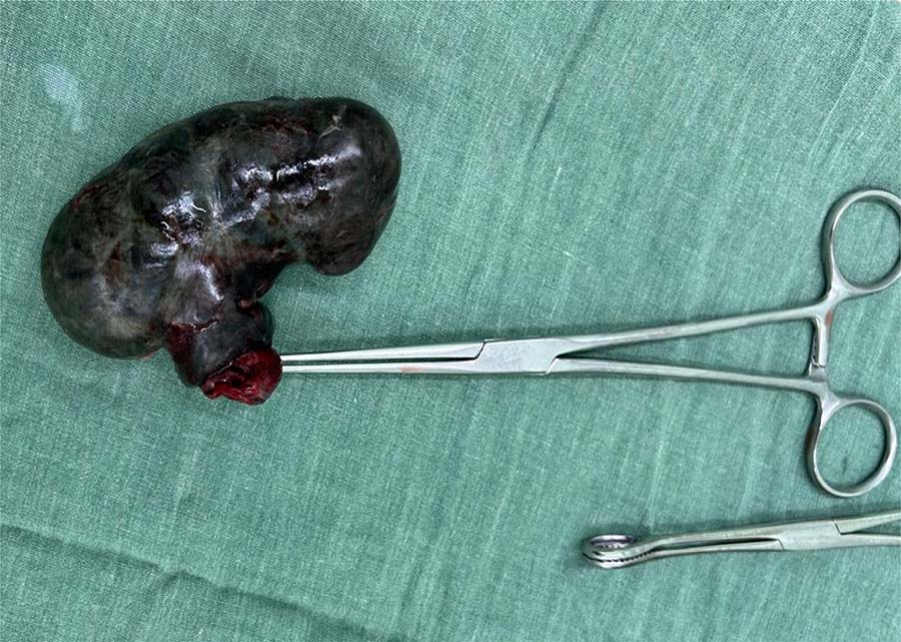
Post diverticulectomy.

**Figure 4:**
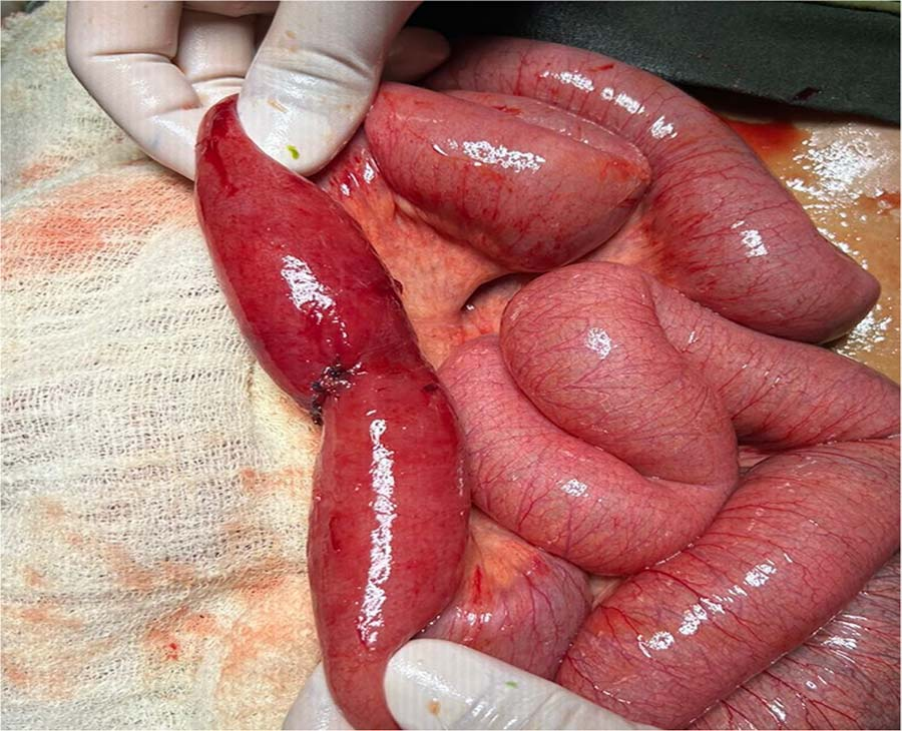
Wedge resection performed.

Intraoperatively, a Meckel’s diverticulum measuring ~6 cm in diameter was identified 60 cm proximal to the ileocecal valve (Fig. 1). A fibrous band originating at the diverticular base acted as a point of fixation, resulting in axial torsion and irreversible vascular compromise of the diverticulum (Fig. 2). No evidence of appendiceal pathology was identified. A diverticulectomy with wedge resection was performed, followed by primary two-layer closure using 3–0 Vicryl sutures (Figs 3 and 4). The procedure was completed without complications.

The postoperative course was favorable. The patient remained nil per os for 72 hours, after which oral intake was gradually resumed. Bowel function recovered adequately, and diet was advanced as tolerated. He was discharged on postoperative Day 4 with oral antibiotics and analgesics. Follow-up evaluation demonstrated complete recovery without complications.

## Discussion

Meckel’s diverticulum is classified histologically as a true diverticulum because it contains all layers of the intestinal wall [8]. Despite being the most common congenital anomaly of the small intestine, symptomatic disease remains uncommon, particularly in adults [1]. As a result, diagnosis is frequently delayed and often established only after surgical exploration [9].

The clinical presentation of complicated Meckel’s diverticulum overlaps considerably with other causes of acute abdomen. In adults, intestinal obstruction and diverticulitis are the most frequently reported complications, whereas gastrointestinal bleeding predominates in children [6, 10, 11]. In the present case, the patient presented with diffuse abdominal pain, leukocytosis, and generalized peritoneal irritation, findings highly suggestive of complicated appendicitis. Consequently, the correct diagnosis was only achieved intraoperatively.

Axial torsion represents one of the rarest complications of Meckel’s diverticulum. The condition occurs when the diverticulum rotates around its own axis, compromising venous and arterial blood flow and eventually leading to ischemia and gangrene. Several anatomical factors have been implicated in its development, including excessive diverticular length, a narrow diverticular base, and the presence of mesodiverticular or fibrous bands. These bands, remnants of embryologic structures, may create a fixed point around which torsion occurs [12].

In the presented patient, the fibrous band identified at surgery appeared to be the principal factor responsible for torsion and subsequent vascular compromise. The resulting ischemic changes explained the acute presentation and signs of peritonitis. Similar cases described in the literature emphasize that torsion should be considered when a gangrenous Meckel’s diverticulum is encountered during surgery for suspected appendicitis or bowel obstruction.

The optimal management of symptomatic Meckel’s diverticulum is surgical resection. The choice between simple diverticulectomy, wedge resection, or segmental bowel resection depends on the diverticular anatomy, the condition of the adjacent ileum, and the presence of ischemia, perforation, or ectopic tissue. In the present case, wedge resection allowed complete removal of the compromised diverticulum while preserving healthy bowel.

This case highlights the importance of maintaining a broad differential diagnosis in young adults presenting with acute abdomen. Although symptomatic Meckel’s diverticulum is uncommon, rare complications such as axial torsion can result in rapid clinical deterioration and require urgent surgical treatment. Early operative intervention remains essential for definitive diagnosis and favorable outcomes.

## Data Availability

The data and images supporting the findings of this case report are not publicly available due to patient privacy restrictions.
